# Cost of treatment of metastatic non-small lung cancer in Sweden, 2011–2023

**DOI:** 10.2340/1651-226X.2025.44650

**Published:** 2025-10-26

**Authors:** Kun Kim, Michael Sweeting, Nils Wilking, Linus Mattias Jönsson

**Affiliations:** a Department of Neurobiology, Care Sciences and Society, Karolinska Institutet, Stockholm, Sweden; b Nordic HTA, AstraZeneca AB, Stockholm, Sweden; c Statistical Innovation, AstraZeneca, UK; d Department of Oncology-Pathology, Karolinska Institutet, Stockholm, Sweden

**Keywords:** Metastatic non-small cell lung cancer, immune-oncology, EGFR-targeted therapy, ALK-targeted therapy, healthcare resource use, costs

## Abstract

**Background and purpose:**

Metastatic non-small cell lung cancer (mNSCLC) contributes to the economic burden. Over the past decade, treatment has evolved with the introduction of epidermal growth factor receptor (EGFR) anaplastic lymphoma kinase (ALK)-targeted, and immune-oncology (IO) drugs. However, limited evidence exists on the long-term costs of mNSCLC treatments in Sweden.

**Patient/material and methods:**

This population-based retrospective study used data from the National Board of Health and Welfare, identifying patients initially diagnosed with stage IV NSCLC between 2011 and 2020. Healthcare costs, including inpatient care, outpatient care, and drug expenses, were assessed using Diagnosis-Related Group (DRG) tariffs and prescription data. Drug expenses exceeding DRG tariff limits, such as IO drugs, were calculated separately based on retail list prices. Costs were analyzed over 5 years post-diagnosis and adjusted to 2023 values.

**Results:**

A total of 17,107 patients were included. IO drug use increased sharply after 2016, becoming the predominant therapy. EGFR- and ALK-targeted drug use steadily increased. Overall costs rose over time, especially in the first year after diagnosis. The first-year mean cost was highest among patients receiving IO drugs (€105,286), primarily due to drug acquisition, but declined in subsequent years. ALK- and EGFR-targeted therapies also had high initial costs but remained stable thereafter.

**Interpretation:**

This study highlights the increasing economic burden of mNSCLC treatment in Sweden, driven by the targeted and IO drugs. While ALK-, EGFR-targeted, and IO drugs contribute to high first-year mean costs, IO drug costs decline significantly in subsequent years after diagnosis.

## Introduction

Lung cancer is one of the most common cancers in Sweden, with approximately half of new cases diagnosed with metastasis, often resulting in poor treatment outcomes and high mortality rates [1]. Alongside its severe disease burden, lung cancer imposes a substantial economic burden. Lung cancer incurred the highest economic burden among all cancer types, with total costs of €18.8 billion, accounting for 15% of overall cancer-related expenses in Europe. These costs include healthcare resource use, productivity loss, and informal care [2]. In the UK, the annual costs of healthcare resource use per lung cancer patient was £9,071, significantly higher than other common cancers [3]. The costs associated with lung cancer may vary across countries due to differences in the healthcare delivery system. In Sweden, the annual health expenditure on cancer care was estimated at €1.9 billion, accounting for 3.7% of the total healthcare expenditure, with €572 million allocated to cancer drugs [4]. However, no recent studies in literature report a detailed breakdown of costs per lung cancer patient by different types of healthcare resource use in Sweden.

Over the past decade, the treatment landscape for metastatic non-small cell lung cancer (mNSCLC) has undergone significant changes due to advances in therapeutic options. Targeted therapies have been introduced for patients with specific genetic mutations including epidermal growth factor receptor (EGFR) and anaplastic lymphoma kinase (ALK). Since the approval of the first-generation EGFR inhibitor in mNSCLC in 2009 [5], a series of EGFR-targeted therapies have been introduced in Sweden. Osimertinib, as the third-generation EGFR inhibitor, was initially approved for T790M-positive mNSCLC and later expanded to first-line treatment in 2018 [6]. Similarly, since crizotinib became the first ALK inhibitor approved in 2012 [7], the development of newer ALK inhibitors continued, adding EMA approval of lorlatinib in 2019 for advanced ALK-positive NSCLC after the failure of other ALK inhibitors, as well as alectinib in 2017 for second-line and in 2020 for first-line treatment [8]. These mutations are present in approximately 10–15% (among Caucasian patients) and 5%, retrospectively, of mNSCLC patients [9, 10]. For the broader population of mNSCLC patients without these mutations, PD-(L)1 inhibitors were initially granted EMA approval as second-line treatment in 2015, and the indication was expanded to first-line treatment for patients with high PD-L1 expression [11, 12]. Over time, their indication has gradually broadened to include all PD-L1-positive patients and, more recently, PD-L1-negative patients eligible for immune-oncology therapies (IO) [13]. Although these novel therapies have shown significant survival benefits, their high acquisition costs raised financial concerns during the health technology assessment in Sweden [14–19].

Introduction of the novel therapies has likely led to higher drug costs in the early years following diagnosis compared to chemotherapy (CT) drugs alone. However, it remains unclear whether these elevated drug costs would have persisted or diminished in the later years. The benefits of improved disease management may have reduced burden on healthcare services, potentially, affecting overall healthcare resource use. Conversely, extended survival might have resulted in prolonged resource consumption that would otherwise have been avoided due to immature mortality. Despite these uncertainties due to the widening adoption of these novel therapies, a significant evidence gap remains regarding costs of healthcare resource use in treatment of mNSCLC patients in Sweden.

This analysis aimed to describe the expansion of novel anti-cancer therapy use from 2011 to 2020 and assess its impact on costs of treating mNSCLC patients over the follow-up years after diagnosis. We categorized overall costs by type of healthcare resource use into inpatient care, outpatient care, other prescribed drugs as well as anti-cancer therapy drugs.

### Patients/material and methods

We conducted a population-based retrospective study using data from the National Board of Health and Welfare (NBHW) in Sweden. The NBHW oversees healthcare records of cancer patients, in collaboration with the national quality registries. Healthcare providers are mandated to report all new cancer cases, including demographic characteristics, diagnosis, and histology, as these data are known for high completeness [20]. However, details obtained through clinical examinations, laboratory tests, biomarker tests and CT administration are not mandatory. Patients with stage IV NSCLC at diagnosis were identified from the national lung cancer register between January 1, 2011 and December 31, 2020. Patients who were initially diagnosed with early-stage disease and later progressed to stage IV were not included, as this could not be captured within the analysis dataset, although inclusion would have provided a more comprehensive assessment. Healthcare resource use of these patients was followed until death or the end date of the study on December 31, 2023.

The cancer patient records are linked to the national patient registry, which provides resource uses during inpatient and outpatient care, including admission and discharge dates, outpatient visits, diagnosis codes, operation codes, and Diagnosis-Related Group (DRG) codes. Costs for inpatient and outpatient care were calculated using DRG codes, corresponding DRG tariffs for the relevant year [21]. This DRG-based approach facilitates a comprehensive assessment of healthcare resource utilization, aligning costs with the complexity and resource requirements of healthcare services. Additionally, it provides an average cost estimate, which is representative of national healthcare practices [22]. Since DRG codes for 2023 were not yet assigned, we used a local DRG grouper to allocate appropriate DRG codes based on these patient records. Costs of inpatient and outpatient care were categorized further as cancer-related and non-cancer-related costs using specific DRG codes as detailed in Supplementary Table 1.

Costs of oral drugs including anti-cancer drugs such as ALK- and EGFR-targeted drugs and oral CT drugs, immunosuppressants, and non-cancer-related drugs were calculated using the prescription data. The data contained product name, ATC code, and total cost per patient including co-payments, extracted from the national prescription registry. Costs of intravenous (IV) drugs were calculated using DRG tariffs, such as R51O (CT administration during outpatient visit), which had a 2023 tariff of SEK 8,598, including costs of acquiring CT drugs. These DRG tariffs exclude outlier cases where costs exceed the upper limit. Thus, as costs per dose of IO drugs exceed this limit, that is, SEK 34,040, these drug costs were accounted for separately. Drug use records of the IO drugs were extracted from the Individual Patient Overview data (Individuell patientöversikt). The number of cycles administrated for IO drugs was calculated based on treatment durations, defined as the period between the start and end dates, with cycle intervals aligned with those reported in the trials or the summary of product characteristics (SmPC). For 9.7% of the patients treated with IO drugs who lacked end dates of drug administration, the median number of cycles from the trials was assumed [11, 12, 23–25]. The drug costs were calculated based on the retail list prices, including VAT. However, the actual prices paid may be lower, since hospital drugs can be procured under confidential discount agreements.

Patients were categorized into three cohorts based on diagnosis date: 2011–2013, 2014–2016, and 2017–2020, to analyze cost trends over the past decade. Additionally, patients were stratified into four cohorts based on the administration of at least one dose of EGFR-targeted drugs, ALK-targeted drugs, IO drugs, or CT drugs alone without combination of these novel drugs within the first year after diagnosis. These cohorts were not mutually exclusive, as crossover between drug categories is common in clinical practice. For mNSCLC patients, IO drugs are often administered in combination with platinum-based CT while for the patients with EGFR or ALK mutations, the recommended therapies are EGFR- or ALK-targeted drugs without CT. Before the introduction of these novel drugs over the last years, CT drugs alone were also used as a first line therapy. CT drugs alone remains an option as a second or subsequent line of therapies. For the patients who initially received EGFR- or ALK-targeted drugs, IO drugs became available as a secondary therapeutic option following expanded indications for IO [26, 27]. To reflect real-world clinical practice patterns, patients who initially received drugs from one category but later switched to another were included in the respective cohorts. The CT drugs alone cohort included patients who only received CT drugs within the first year after diagnosis although some later received one of the novel drugs. The drug names and their ATC codes used for the cohort stratification are presented in Supplementary Table 2.

Costs were calculated from the date of diagnosis and divided into 12-month intervals up to 5 years post-diagnosis. Mean overall healthcare costs were estimated using the number of patients at risk who were alive and not censored at the beginning of each follow-up year. All costs were adjusted to 2023 values using the consumer price index for the health sector when applicable [28], and presented in Euros using an exchange rate of EUR 1 = SEK 11.48 [29].

Ethical approval was obtained from the Swedish Ethical Review Authority. Individual consent was not required as the data were anonymized. The analyses were performed using R version 4.2.3.

## Results

**Figure 1:**
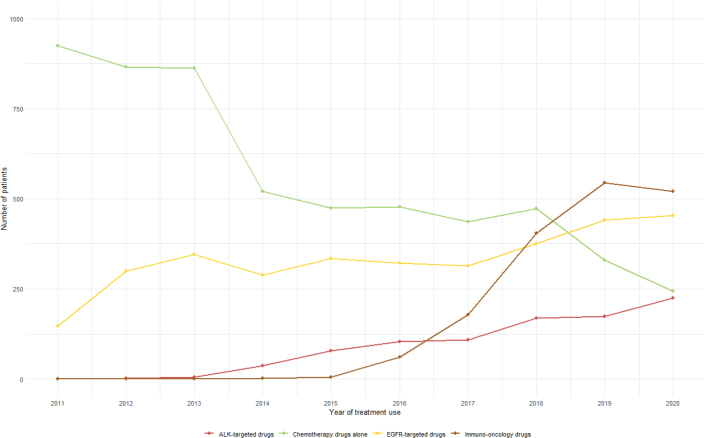
Trends in the number of patients receiving different types of anti-cancer therapy, 2011–2020.

A total of 17,107 patients who were initially diagnosed with stage IV NSCLC between 2011 and 2020 were included. Figure 1 presents the number of patients using different types of anti-cancer drugs, clearly illustrating the shifting treatment patterns for mNSCLC over the last decade. CT drugs alone initially had the highest number of patients, but their use declined through the period. In contrast, IO drugs began to rise sharply around 2016, surpassing CT drugs alone by 2019. EGFR-targeted drug use steadily increased over the years, while ALK-targeted drugs remained at the lowest level compared to other drug types but showed a gradual increase since 2013.

**Figure 2:**
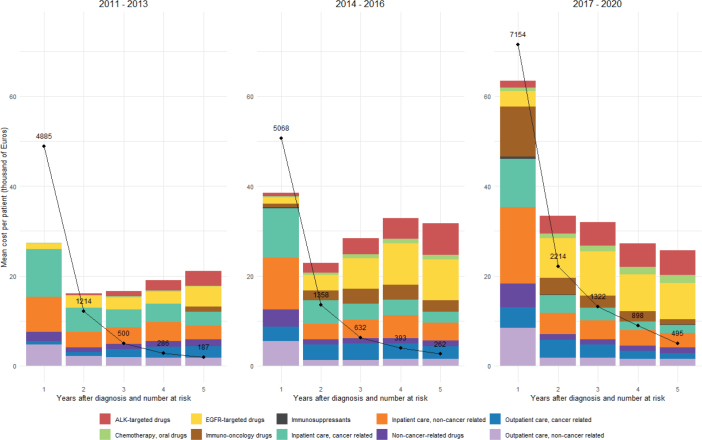
Mean cost per patient at risk at the start of each follow-up year by time period (in 1,000 Euros). *The black dots and lines represent the number of patients at risk at the start of each follow-up year.

Figure 2 presents the mean cost per patient at risk at the start of each follow-up year across three different time periods: 2011–2013, 2014–2016, and 2017–2020. A noticeable trend is the increase in the overall costs over time, particularly in the first year following diagnosis. In 2011–2013, the first-year mean cost was relatively moderate, whereas in 2017–2020, there was a substantial rise in the first-year cost, mainly driven by increased spending on IO drugs and non-cancer-related inpatient care. While the increase in IO drug costs reflects the expanded use of IO therapies over the last decade, the rise in non-cancer-related inpatient costs is partly due to the nearly doubled DRG weights over the same period, despite the DRG point values remaining relatively stable.

In 2017–2020, the mean costs tended to decrease in subsequent years whereas in 2011–2013 and 2014–2016, the mean costs from the second to fifth year tended to increase. This pattern likely reflects the growing use of novel therapies as subsequent treatment options. Inpatient care costs, both cancer-related and non-cancer-related, remained a significant component throughout all time periods, although their relative share varied. A data legend presenting the detailed outcomes is available in Supplementary Table 3.

Table 1 presents the total costs of €607 million over 5 years post diagnosis in the 2017–2020 cohort (*N* = 7,154). The largest contributors were inpatient care, with cancer-related and non-cancer-related hospitalizations accounting for 38.5% of total costs. Both cancer-related and non-cancer-related outpatient care added a further 19.2%. Among drug costs, IO accounted for 15.4% of the total, followed by EGFR-targeted (11.3%) and ALK-targeted drugs (5.6%).

**Table 1:** The total costs over 5 years post diagnosis in the 2017–2020 cohort in Euros (million).

	Year 1	Year 2	Year 3	Year 4	Year 5	Total
***2017–2020 (N = 7,154)***						
*Cost by healthcare resource type (€ million)*						
ALK-targeted drugs	10.9	8.7	6.9	4.7	2.8	34.0
EGFR-targeted drugs	24.7	19.6	13.1	7.4	4.0	68.8
Immuno-oncology drugs	79.3	8.3	3.4	2.0	0.6	93.6
Immunosuppressants	4.3	0.1	0.1	0.0	0.1	4.6
Inpatient care, cancer related	76.4	8.9	3.6	1.7	0.9	91.6
Inpatient care, non-cancer related	122.0	10.4	5.6	3.2	1.6	142.0
Outpatient care, cancer related	32.7	8.7	3.8	1.6	0.6	47.5
Outpatient care, non-cancer related	60.5	4.1	2.3	1.4	0.8	69.1
Chemotherapy, oral drugs	5.2	2.2	1.7	1.4	0.9	11.5
Non-cancer-related drugs	37.7	2.8	1.6	1.0	0.6	43.8
Total	453.0	73.8	42.2	24.5	12.7	607.0

There were 8,523 patients who received CT drugs alone (*N* = 5,073), IO drugs (*N* = 1,436), EGFR- (*N* = 1,647), or ALK-targeted drugs (*N* = 367) within the first year after diagnosis. A total of 3,384 patients were recorded as having no active treatments planned. Table 2 presents baseline patient characteristics of these patients. The CT drugs alone cohort was generally older and had a lower proportion with performance status 0–1. The IO drugs cohort were slightly older with a larger proportion of squamous carcinoma compared to the other treatment cohorts. The EGFR- and ALK-targeted cohorts were younger with the highest proportion of adenocarcinoma and never-smokers.

**Table 2:** Baseline patient characteristics by types of anti-cancer therapy.

*N*	Chemotherapy drugs alone	EGFR-targeted drugs	ALK-targeted drugs	Immuno-oncology drugs	No active treatments planned
	5,073	1,647	367	1,436	3,384
Mean age (SD)	68.63 (8.66)	67.61 (10.91)	61.85 (13.64)	69.06 (8.60)	75.83 (8.76)
Sex = Male (%)	2,529 (49.9)	601 (36.5)	170 (46.3)	683 (47.6)	1,775 (52.5)
Histology					
Adenocarcinoma	3,529 (69.6)	1,505 (91.4)	343 (93.5)	1,022 (71.2)	2,145 (63.4)
Adenosquamous	40 (0.8)	8 (0.5)	2 (0.5)	17 (1.2)	32 (0.9)
Large cell	531 (10.5)	63 (3.8)	6 ( 1.6)	74 (5.2)	415 (12.3)
Other	97 (1.9)	16 (1.0)	6 ( 1.6)	29 (2.0)	178 (5.3)
Squamous	872 (17.2)	55 (3.3)	10 ( 2.7)	294 (20.5)	614 (18.1)
Performance status					
0	1,060 (20.9)	484 (29.4))	136 (37.1)	370 (25.8)	49 (1.4)
1	2,412 (47.5)	734 (44.6)	132 (36.0)	766 (53.3)	239 (7.1)
2	1,344 (26.6)	302 (18.3)	74 (20.2)	246 (17.1)	593 (17.5)
3	225 (4.4)	112 ( 6.8)	21 ( 5.7)	47 (3.3)	1,588 (46.9)
4	21 (0.4)	12 ( 0.7)	3 ( 0.8)	4 (0.3)	759 (22.4)
Unknown	11 (0.2)	3 (0.2)	1 ( 0.3)	3 (0.2)	156 (4.6)
Smoking					
Former smoker	2,475 (48.8)	704 (42.7)	139 (37.9)	760 (52.9)	1,638 (48.4)
Never smoker	505 (10.0)	657 (39.9)	165 (45.0)	151 (10.5)	430 (12.7)
Smoker	2,070 (40.8)	277 (16.8)	62 (16.9)	522 (36.4)	1,255 (37.1)
Unknown	23 (0.5)	9 (0.5)	1 ( 0.3)	3 (0.2)	61 (1.8)
Site					
Adrenal gland	427 (8.4)	59 ( 3.6)	15 ( 4.1)	124 (8.6)	242 (7.2)
CNS	672 (13.2)	159 ( 9.7)	36 ( 9.8)	117 (8.1)	257 (7.6)
Liver	373 (7.4)	96 (5.8)	38 (10.4)	108 (7.5)	423 (12.5)
Other	1,462 (28.8)	436 (26.5)	70 (19.1)	205 (14.3)	898 (26.5)
Pleura	252 (5.0)	132 ( 8.0)	48 (13.1)	188 (13.1)	374 (11.1)
Bone	1,641 (32.3)	703 (42.7)	144 (39.2)	484 (33.7)	1,025 (30.3)
Unknown	246 (4.8)	62 ( 3.8)	16 ( 4.4)	210 (14.6)	165 (4.9)
Death = TRUE (%)	4,926 (97.1)	1,516 (92.0)	257 (70.0)	1,206 (84.0)	3,375 (99.7)

* The cohorts were not mutually exclusive. A total of 26 patients received both EGFR-targeted and IO drugs, 29 patients received both ALK-targeted and IO drugs, and 3 patients received both EGFR- and ALK-targeted drugs.

** CNS: Central Nervous System.

**Figure 3:**
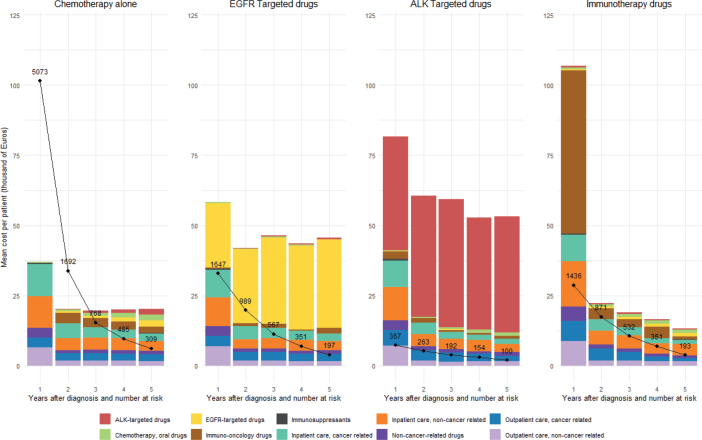
Mean cost per patient at risk at the start of each follow-up year by type of anti-cancer therapy (in 1,000 Euros). *The black dots and lines represent the number of patients at risk at the start of each follow-up year.

Figure 3 shows the mean cost per patient at risk at the start of each follow-up year by treatment cohort. The IO drugs cohort had the highest first-year cost of €106,707, largely attributable to the IO drug expenses, but costs declined markedly thereafter, reaching €13,342 in the fifth year. The EGFR- and ALK-targeted drugs cohorts also had a high first-year cost, which then remained relatively stable over follow-up. The CT drugs alone cohort showed the lowest first-year cost with a similar stable pattern in later years. A data legend presenting the detailed outcomes is available in Supplementary Table 4.

## Discussion and conclusion

The study illustrated the increasing trend of using the molecularly targeted and IO drugs for treating mNSCLC over the last decade in Sweden, and the overall costs in these patients. Overall costs rose significantly during the 2017–2020 period, largely driven by inpatient care and outpatient care costs, along with the increased use of IO drug in the first year after diagnosis. The highest first-year mean cost was observed among IO drug patients. However, their mean costs significantly dropped in the following years, unlike the other groups where the mean costs in subsequent years remained relatively stable. The clinical trials have shown that IO drugs are administered for a median duration of less than 1 year [30–34], whereas EGFR- and ALK-targeted drugs are continued for more than few years in responding patients [35–38]. Our findings confirm that these expected patterns from clinical trials are also observed in real-world clinical practice.

This study highlights the economic impact of introducing novel therapies by providing a detailed assessment of the costs associated with mNSCLC treatment over the past decade. The comprehensive cost breakdown by healthcare resource type offers understanding of key cost drivers, supporting informed decision-making regarding the introduction of novel drugs. By identifying the higher upfront costs of these therapies and their potential for extended survival, the study underscores the importance of evolving treatment patterns and resource utilization. It also underscores the need to adopt a broader perspective in evaluating economic value of cancer care, one that considers not only cancer-specific treatments but also the substantial costs associated with non-cancer-related healthcare services. The analysis was conducted from the healthcare system perspective restricted to direct medical costs related to inpatient care, outpatient care, and drug acquisition. Indirect costs, such as productivity losses or informal care, were not included. Notably, the rising costs of IO drugs reflect their expanded use over time, while the increase in other expenses, particularly non-cancer-related inpatient costs, has been influenced by structural changes in the reimbursement system, including a near doubling of DRG weights over the past decade. This shift is important to consider when interpreting cost differences across time periods, as well as between treatment types such as IO and CT, given that IO patients are more likely to have been treated in recent years.

Although direct comparisons are challenging, our findings appeared to be similar to previous studies in Nordic countries. Based on hospital register data in Finland, treatment costs including in-patient and out-patient care costs as well as medication expenses for the first 24 months were estimated at €22,000 in 2016 and €26,000 in 2017 [39]. These estimates reflect a period before the reimbursement of first-line osimertinib, with only partial use in the second line, and at the very beginning of IO therapy in Finland. Nevertheless, these estimates remained lower than our results reporting first and second year follow-up costs in the 2014–2016 cohort. According to Comparator Report on Cancer in Europe 2025, uptake of medicines in lung cancer in Finland is nearly half that of Sweden, which may explain the lower cost estimates [40]. This might be also due to the study design that relied on activity-based costing method considering hospitalization lengths, number of outpatient visits, medication doses, etc. whereas our study comprehensively approached with DRG tariffs, and the inflation factor over the last decade that the Finnish study did not apply. Gouliaev et al. 2021 estimated the direct annual cost of lung cancer, regardless of stage, at €15,026 per person for the period 1998–2010, based on population-based registry data in Denmark [41]. In our study, the average cost weighted by the numbers at risk each year was €24,161 for the 2011–2013 cohort. Considering the early-stage lung cancer patients likely contributing to lower costs and the inflation factor not applied in the Danish study, the estimate may appear comparable to our results.

This study has several limitations that should be considered when interpreting the findings. First, the analysis did not account for potential regional or hospital-specific differences in prescribing practices or drug availability. In Sweden, national assessments are made with recommendations passed to the regions, which decide on adoption and financing for hospital-administered drugs. For oral drugs, reimbursement decisions are made nationally, regulating access and patient costs. Clinical practice is guided by the National Lung Cancer Care Program, which oncologists are expected to follow. The results reflect national averages and may not capture local variation that arises from differences in clinical decision-making, treatment practices, and the pace of adoption of new cancer therapies. Such variability could introduce biases in estimating the total costs of care. Second, the study did not capture costs incurred before diagnosis or assess potential variations in hospital to account for diagnostic practices. These gaps may affect the comprehensiveness and comparability of cost estimates across cohorts. Third, the study did not assess any survival differences between the cohorts by drug type since it was not designed to evaluate survival outcomes. Fourth, the analysis was limited to direct costs of healthcare resource use and excluded indirect costs, such as productivity losses, caregiver burden, and societal impacts. Bugge et al. reported the fact that for lung cancer patients, 51% of total costs were attributable to production losses due to premature death, 12% to work absenteeism, and only 37% to patient-related hospital treatment costs in Norway. The proportion of indirect costs for lung cancer was notably higher than the average of all cancers [42]. Fifth, repayments may be arranged between the pharmaceuticals and the regional authorities in Sweden, which effectively function as price discounts [43]. IO drugs and some of EGFR targeted drugs included in this analysis are subject to these confidential price agreements [44]. Similar price arrangements exist in other European countries, with reported discounts ranging from 0 to 50% [45]. However, the lack of information on these discounts limits the interpretation of the results for the actual expenses on these drugs in practice. Sixth, we did not construct separate cohorts for rare molecular alterations such as ROS1 or RET because of their low incidence in mNSCLC and the later approval and uptake within or after our study inclusion window. This limited both observation time and their contribution to understanding the overall cost of treating mNSCLC in Sweden. Nevertheless, including such patients in future studies may help provide a more comprehensive overview of the evolving economic burden as these therapies become more established. Finally, home-based healthcare services were not included in this analysis. Bergqvist et al. reported that specialized home-based palliative care services reduced emergency department visits by 51% and hospitalizations by 41% in Sweden [46]. Considering the diseases severity of mNSCLC, the inclusion of costs of such care could be relevant in the analysis.

This study highlights the significant cost burden of treating mNSCLC in Sweden, with an increasing trend following the introduction of the molecularly targeted and IO drugs. The findings demonstrate that novel therapies, such as ALK- and EGFR-targeted drugs and IO drugs, contribute to higher costs, especially in the first year following diagnosis, while the costs of IO drugs significantly decrease in subsequent years. Addressing data limitations and incorporating broader societal costs in future studies will enhance the understanding of the full economic impact of mNSCLC treatments.

## Supplementary Material



## Data Availability

The data used in this study are not publicly available, as they were obtained from the Swedish National Board of Health and Welfare under an agreement that restricts public access. Access to the data was granted for research purposes only.
