# Supplementary material for: Cost of treatment of metastatic non-small lung cancer in Sweden, 2011–2023
Source: Acta Oncol. 2025 Oct 26;64:44650. doi: 10.2340/1651-226X.2025.44650 (PMC12576694; doi:10.2340/1651-226X.2025.44650)
Supplement: Supplementary file 1 [file AO-64-44650-s1.pdf]

Supplementary material has been published as submitted. It has not been copyedited, or typeset by Acta Oncologica

Supplemental table 1 DRG codes, specifying cancer related treatment

DRG	text	text translated to English
A03A	Intrakraniell kir för tumör M	Intracranial surgery for tumor with severe complication
A03E	Intrakraniell kir för tumör U	Intracranial surgery for tumor without complication
A16N	Intrakraniell strålbehandling	Intracranial radiotherapy
A16O	Intrakraniell strålbehandling O	Intracranial radiotherapy O
A43A	Tumörer i nervsyst M	Tumors in nervous system with severe complication
A43C	Tumörer i nervsyst K	Tumors in nervous system with mild complication
A43E	Tumörer i nervsyst U	Tumors in nervous system without complication
A43O	Tumörer i nervsyst O	Tumors in nervous system
A43P	Läk tumörer i nervsyst K O	Physician visit for tumors in nervous system with mild complication
A43Q	Läk tumörer i nervsyst U O	Physician visit for tumors in nervous system without complication
B29N	Lokal strålbehandling öga	Local radiotherapy for the eye
C30A	Mal/oklar tum öra näs mun hals M	Malignant/unclear tumor ENT with severe complication
C30C	Mal/oklar tum öra näs mun hals K	Malignant/unclear tumor ENT with mild complication
C30E	Mal/oklar tum öra näs mun hals U	Malignant/unclear tumor ENT without complication
C30O	Läk mal/oklar tumör ÖNH O	Physician visit for malignant/unclear tumor ENT
D35A	Tumörer i andningsorganen M	Tumors in respiratory organs with severe complication
D35C	Tumörer i andningsorganen K	Tumors in respiratory organs with mild complication
D35E	Tumörer i andningsorganen U	Tumors in respiratory organs without complication
D35O	Läk tumörer i andningsorg O	Physician visit for tumors in respiratory organs
F39A	Malign tumör mage tarm M	Malignant tumor stomach intestines with severe complication
F39C	Intrakraniell kir för tumör M	Intracranial surgery for tumor with severe complication
F39E	Intrakraniell kir för tumör U	Intracranial surgery for tumor without complication
F39O	Intrakraniell strålbehandling	Intracranial radiotherapy
F39P	Intrakraniell strålbehandling O	Intracranial radiotherapy O
F39Q	Tumörer i nervsyst M	Tumors in nervous system with severe complication
G20A	Tumörer i nervsyst K	Tumors in nervous system with mild complication
G20E	Tumörer i nervsyst U	Tumors in nervous system without complication
G33A	Tumörer i nervsyst O	Tumors in nervous system
G33C	Läk tumörer i nervsyst K O	Physician visit for tumors in nervous system with mild complication
G33E	Läk tumörer i nervsyst U O	Physician visit for tumors in nervous system without complication
G33O	Lokal strålbehandling öga	Local radiotherapy for the eye
K02N	Mal/oklar tum öra näs mun hals M	Malignant/unclear tumor ENT with severe complication
K02O	Mal/oklar tum öra näs mun hals K	Malignant/unclear tumor ENT with mild complication
K04N	Mal/oklar tum öra näs mun hals U	Malignant/unclear tumor ENT without complication
K04O	Läk mal/oklar tumör ÖNH O	Physician visit for malignant/unclear tumor ENT
K20C	Tumörer i andningsorganen M	Tumors in respiratory organs with severe complication
K20E	Tumörer i andningsorganen K	Tumors in respiratory organs with mild complication

K20O	Tumörer i andningsorganen U	Tumors in respiratory organs without complication
M05A	Läk tumörer i andningsorg O	Physician visit for tumors in respiratory organs
M05C	Malign tumör mage tarm M	Malignant tumor stomach intestines with severe complication
M05E	Intrakraniell kir för tumör M	Intracranial surgery for tumor with severe complication
M35A	Intrakraniell kir för tumör U	Intracranial surgery for tumor without complication
M35C	Intrakraniell strålbehandling	Intracranial radiotherapy
M35E	Intrakraniell strålbehandling O	Intracranial radiotherapy O
M35O	Tumörer i nervsyst M	Tumors in nervous system with severe complication
N10C	Tumörer i nervsyst K	Tumors in nervous system with mild complication
N10E	Tumörer i nervsyst U	Tumors in nervous system without complication
N30C	Tumörer i nervsyst O	Tumors in nervous system
N30E	Läk tumörer i nervsyst K O	Physician visit for tumors in nervous system with mild complication
N40C	Läk tumörer i nervsyst U O	Physician visit for tumors in nervous system without complication
N40E	Lokal strålbehandling öga	Local radiotherapy for the eye
N40O	Mal/oklar tum öra näs mun hals M	Malignant/unclear tumor ENT with severe complication
O02O	Mal/oklar tum öra näs mun hals K	Malignant/unclear tumor ENT with mild complication
O10O	Mal/oklar tum öra näs mun hals U	Malignant/unclear tumor ENT without complication
O28C	Läk mal/oklar tumör ÖNH O	Physician visit for malignant/unclear tumor ENT
O28E	Tumörer i andningsorganen M	Tumors in respiratory organs with severe complication
O40A	Tumörer i andningsorganen K	Tumors in respiratory organs with mild complication
O40C	Tumörer i andningsorganen U	Tumors in respiratory organs without complication
O40E	Läk tumörer i andningsorg O	Physician visit for tumors in respiratory organs
O40O	Malign tumör mage tarm M	Malignant tumor stomach intestines with severe complication
O40P	Intrakraniell kir för tumör M	Intracranial surgery for tumor with severe complication
O40Q	Intrakraniell kir för tumör U	Intracranial surgery for tumor without complication
P40O	Intrakraniell strålbehandling	Intracranial radiotherapy
R35N	Intrakraniell strålbehandling O	Intracranial radiotherapy O
R35O	Tumörer i nervsyst M	Tumors in nervous system with severe complication
R36C	Tumörer i nervsyst K	Tumors in nervous system with mild complication
R36E	Tumörer i nervsyst U	Tumors in nervous system without complication
R36O	Tumörer i nervsyst O	Tumors in nervous system
R40C	Läk tumörer i nervsyst K O	Physician visit for tumors in nervous system with mild complication
R40E	Läk tumörer i nervsyst U O	Physician visit for tumors in nervous system without complication
R50N	Lokal strålbehandling öga	Local radiotherapy for the eye
R50O	Mal/oklar tum öra näs mun hals M	Malignant/unclear tumor ENT with severe complication
R51N	Mal/oklar tum öra näs mun hals K	Malignant/unclear tumor ENT with mild complication
R51O	Mal/oklar tum öra näs mun hals U	Malignant/unclear tumor ENT without complication
R60N	Läk mal/oklar tumör ÖNH O	Physician visit for malignant/unclear tumor ENT
R60O	Tumörer i andningsorganen M	Tumors in respiratory organs with severe complication
R65A	Tumörer i andningsorganen K	Tumors in respiratory organs with mild complication
R65C	Tumörer i andningsorganen U	Tumors in respiratory organs without complication
R65E	Läk tumörer i andningsorg O	Physician visit for tumors in respiratory organs
R97O	Malign tumör mage tarm M	Malignant tumor stomach intestines with severe complication

R99O	Intrakraniell kir för tumör M	Intracranial surgery for tumor with severe complication
R99X	Intrakraniell kir för tumör U	Intracranial surgery for tumor without complication
R99Z	Intrakraniell strålbehandling	Intracranial radiotherapy
W20C	Intrakraniell strålbehandling O	Intracranial radiotherapy O
W20E	Tumörer i nervsyst M	Tumors in nervous system with severe complication
W42N	Tumörer i nervsyst K	Tumors in nervous system with mild complication
X10O	Tumörer i nervsyst U	Tumors in nervous system without complication
X11O	Tumörer i nervsyst O	Tumors in nervous system
X12O	Läk tumörer i nervsyst K O	Physician visit for tumors in nervous system with mild complication
X14O	Läk tumörer i nervsyst U O	Physician visit for tumors in nervous system without complication
X15O	Lokal strålbehandling öga	Local radiotherapy for the eye
001A	Mal/oklar tum öra näs mun hals M	Malignant/unclear tumor ENT with severe complication
010	Mal/oklar tum öra näs mun hals K	Malignant/unclear tumor ENT with mild complication
011	Mal/oklar tum öra näs mun hals U	Malignant/unclear tumor ENT without complication
064	Läk mal/oklar tumör ÖNH O	Physician visit for malignant/unclear tumor ENT
082	Tumörer i andningsorganen M	Tumors in respiratory organs with severe complication
172	Tumörer i andningsorganen K	Tumors in respiratory organs with mild complication
173	Tumörer i andningsorganen U	Tumors in respiratory organs without complication
199	Läk tumörer i andningsorg O	Physician visit for tumors in respiratory organs
203	Malign tumör mage tarm M	Malignant tumor stomach intestines with severe complication
257	Intrakraniell kir för tumör M	Intracranial surgery for tumor with severe complication
258	Intrakraniell kir för tumör U	Intracranial surgery for tumor without complication
259	Intrakraniell strålbehandling	Intracranial radiotherapy
260	Intrakraniell strålbehandling O	Intracranial radiotherapy O
274	Tumörer i nervsyst M	Tumors in nervous system with severe complication
275	Tumörer i nervsyst K	Tumors in nervous system with mild complication
303	Tumörer i nervsyst U	Tumors in nervous system without complication
318	Tumörer i nervsyst O	Tumors in nervous system
319	Läk tumörer i nervsyst K O	Physician visit for tumors in nervous system with mild complication
338	Läk tumörer i nervsyst U O	Physician visit for tumors in nervous system without complication
344	Lokal strålbehandling öga	Local radiotherapy for the eye
346	Mal/oklar tum öra näs mun hals M	Malignant/unclear tumor ENT with severe complication
347	Mal/oklar tum öra näs mun hals K	Malignant/unclear tumor ENT with mild complication
363	Mal/oklar tum öra näs mun hals U	Malignant/unclear tumor ENT without complication
366	Läk mal/oklar tumör ÖNH O	Physician visit for malignant/unclear tumor ENT
367	Tumörer i andningsorganen M	Tumors in respiratory organs with severe complication
406	Tumörer i andningsorganen K	Tumors in respiratory organs with mild complication
407	Tumörer i andningsorganen U	Tumors in respiratory organs without complication
408	Läk tumörer i andningsorg O	Physician visit for tumors in respiratory organs
409	Malign tumör mage tarm M	Malignant tumor stomach intestines with severe complication
410	Intrakraniell kir för tumör M	Intracranial surgery for tumor with severe complication
411	Intrakraniell kir för tumör U	Intracranial surgery for tumor without complication
412	Intrakraniell strålbehandling	Intracranial radiotherapy

413	Intrakraniell strålbehandling O	Intracranial radiotherapy O
414	Tumörer i nervsyst M	Tumors in nervous system with severe complication
465	Tumörer i nervsyst K	Tumors in nervous system with mild complication
492	Tumörer i nervsyst U	Tumors in nervous system without complication
554A	Tumörer i nervsyst O	Tumors in nervous system
003P	Läk tumörer i nervsyst K O	Physician visit for tumors in nervous system with mild complication
258O	Läk tumörer i nervsyst U O	Physician visit for tumors in nervous system without complication
260O	Lokal strålbehandling öga	Local radiotherapy for the eye
407O	Mal/oklar tum öra näs mun hals M	Malignant/unclear tumor ENT with severe complication
408O	Mal/oklar tum öra näs mun hals K	Malignant/unclear tumor ENT with mild complication
410O	Mal/oklar tum öra näs mun hals U	Malignant/unclear tumor ENT without complication
492O	Läk mal/oklar tumör ÖNH O	Physician visit for malignant/unclear tumor ENT
865O	Tumörer i andningsorganen M	Tumors in respiratory organs with severe complication
866O	Tumörer i andningsorganen K	Tumors in respiratory organs with mild complication
867O	Tumörer i andningsorganen U	Tumors in respiratory organs without complication
872O	Läk tumörer i andningsorg O	Physician visit for tumors in respiratory organs
873O	Malign tumör mage tarm M	Malignant tumor stomach intestines with severe complication

Supplemental table 2 Drug names and ATC codes used for cohort stratification

Oral drugs (identified using prescription data)						IV drugs (identified using the National Lung Cancer Register and IPÖ data)	
EGFR-targeted drugs		ALK-targeted drugs		Chemotherapy drugs		Chemotherapy drugs	IO drugs
Names	ATC codes	Names	ATC codes	Names	ATC codes	Names	Names
Afatinib	L01EB03	Alectinib	L01ED03	Cyclophosphamide	L01AA01	Carboplatin	Atezolizumab
Afatinib	L01XE13	Brigatinib	L01ED04	Etoposide	L01CB01	Cisplatin	Durvalumab
Dacomitinib	L01EB07	Ceritinib	L01ED02	Methotrexate	L01BA01	Cyclophosphamide	Ipilimumab
Erlotinib	L01EB02	Crizotinib	L01ED01	Topotecan	L01CE01	Docetaxel	Nivolumab
Erlotinib	L01XE03	Lorlatinib	L01ED05	Vinorelbine	L01CA04	Doxorubicin	Pembrolizumab
Gefitinib	L01EB01					Etoposide	
Gefitinib	L01XE02					Gemcitabine	
Osimertinib	L01EB04					Irinotecan	
Osimertinib	L01XE35					Methotrexate	
						Mitoxantrone	
						Nab-Paclitaxel	
						Paclitaxel	
						Pemetrexed	
						Topotecan	
						Vincristine	
						Vinorelbine	

Note: As all EGFR- and ALK-targeted drugs are orally administered, these patients were identified from prescription data using the ATC codes. Lazertinib gained EMA marketing authorization for NSCLC in November 2024, which falls outside the scope of this study. As all immune-oncology (IO) drugs are intravenously administered, these patients were identified from the National Lung Cancer Register and the Individual Patient Overview data. In 2023, cemiplimab and tremelimumab were also approved for NSCLC in Sweden. Cemiplimab is supplied exclusively through hospital procurement and therefore has no publicly available list price; only two patients received cemiplimab up to the end of 2023 No patients received tremelimumab during the same period. Thus, these two drugs were excluded from this study. Some chemotherapy drugs are available in both intravenous and oral formulations. Patients who were not treated in combination with EGFR-, ALK-targeted or IO drugs were identified from either the National Lung Cancer Register and the IPÖ data or prescription data using the ATC codes.

IO: Immuno-oncology, IPÖ: Individual Patient Overview, IV: intravenous

Supplemental table 3 Mean cost per patient at risk at the start of each follow-up year by time period in Euros

	Year 1	Year 2	Year 3	Year 4	Year 5
2011–2013					
Number of patients at risk	4,885	1,214	500	286	187
Cost by healthcare resource type (€)					
ALK-targeted drugs	41	390	1,235	2,277	3,307
EGFR-targeted drugs	1,290	2,727	2,750	2,654	4,380
Immuno-oncology drugs	-	-	-	-	1,219
Immunosuppressants	63	2	1	10	5
Inpatient care, cancer related	10,547	5,265	3,916	4,155	3,047
Inpatient care, non-cancer related	7,811	3,508	3,768	4,154	3,043
Outpatient care, cancer related	749	888	1,732	2,453	2,680
Outpatient care, non-cancer related	4,710	2,134	1,972	1,802	1,725
Chemotherapy, oral drugs	50	127	172	273	184
Non-cancer-related drugs	2,085	1,061	1,136	1,269	1,510
Total	27,346	16,102	16,682	19,047	21,100
2014–2016					
Number of patients at risk	5,068	1,358	632	393	262
Cost by healthcare resource type (€)					
ALK-targeted drugs	731	2,169	3,621	4,533	7,000
EGFR-targeted drugs	1,385	3,517	6,724	9,277	9,112
Immuno-oncology drugs	799	2,133	3,337	3,305	2,526
Immunosuppressants	280	15	19	3	6
Inpatient care, cancer related	10,964	5,217	3,596	3,406	2,435
Inpatient care, non-cancer related	11,586	3,442	4,012	5,104	3,983
Outpatient care, cancer related	3,182	3,460	3,633	3,373	2,789
Outpatient care, non-cancer related	5,552	1,302	1,335	1,589	1,500
Chemotherapy, oral drugs	249	427	977	1,034	948
Non-cancer-related drugs	3,752	1,171	1,192	1,204	1,347
Total	38,480	22,853	28,446	32,828	31,646
2017–2020					
Number of patients at risk	7,154	2,214	1,322	898	495
Cost by healthcare resource type (€)					
ALK-targeted drugs	1,527	3,933	5,221	5,268	5,551
EGFR-targeted drugs	3,450	8,864	9,905	8,234	8,016
Immuno-oncology drugs	11,083	3,746	2,596	2,248	1,131
Immunosuppressants	600	45	53	31	130
Inpatient care, cancer related	10,685	4,024	2,754	1,901	1,759
Inpatient care, non-cancer related	16,992	4,684	4,236	3,523	3,269
Outpatient care, cancer related	4,577	3,926	2,899	1,784	1,273
Outpatient care, non-cancer related	8,456	1,837	1,774	1,543	1,554
Chemotherapy, oral drugs	731	994	1,319	1,602	1,786
Non-cancer-related drugs	5,267	1,285	1,238	1,121	1,223
Total	63,368	33,338	31,995	27,255	25,692

Supplemental table 4 Mean cost per patient at risk at the start of each follow-up year by type of anti-cancer therapy in Euros

	Year 1	Year 2	Year 3	Year 4	Year 5
Chemotherapy drugs alone					
Number of patients at risk	5,073	1,692	768	485	309
Cost by healthcare resource type (€)					
ALK-targeted drugs	-	217	678	1,173	2,114
EGFR-targeted drugs	-	646	898	1,523	2,167
Immuno-oncology drugs	-	3,523	3,168	2,719	2,267
Immunosuppressants	281	24	63	26	187
Inpatient care, cancer related	11,460	5,453	3,682	3,108	2,636
Inpatient care, non-cancer related	11,320	4,179	4,383	4,197	3,525
Outpatient care, cancer related	3,339	2,591	2,724	2,601	2,367
Outpatient care, non-cancer related	6,586	1,889	1,796	1,786	1,704
Chemotherapy, oral drugs	544	738	1,052	1,707	1,973
Non-cancer-related drugs	3,582	1,084	1,120	1,213	1,280
Total	37,112	20,344	19,564	20,053	20,220
EGFR-targeted drugs					
Number of patients at risk	1,647	989	567	351	197
Cost by healthcare resource type (€)					
ALK-targeted drugs	23	256	410	485	774
EGFR-targeted drugs	23,076	26,452	30,786	29,988	31,439
Immuno-oncology drugs	336	1,056	1,512	473	2,105
Immunosuppressants	321	5	-	-	-
Inpatient care, cancer related	9,986	4,630	3,593	3,186	2,534
Inpatient care, non-cancer related	10,118	3,207	3,735	3,788	3,396
Outpatient care, cancer related	3,665	2,960	3,082	2,762	2,698
Outpatient care, non-cancer related	6,936	1,865	1,787	1,564	1,544
Chemotherapy, oral drugs	300	153	278	271	24
Non-cancer-related drugs	3,545	1,402	1,198	1,083	1,200
Total	58,306	41,986	46,381	43,600	45,714
ALK-targeted drugs					
Number of patients at risk	367	263	192	154	100
Cost by healthcare resource type (€)					
ALK-targeted drugs	40,424	43,100	45,511	39,905	41,376
EGFR-targeted drugs	102	89	407	-	63
Immuno-oncology drugs	2,774	1,693	738	530	1,051
Immunosuppressants	509	22	7	1	1
Inpatient care, cancer related	9,415	4,061	2,314	1,977	1,828
Inpatient care, non-cancer related	11,823	4,276	3,829	3,943	2,842
Outpatient care, cancer related	5,639	3,820	3,222	2,659	1,918
Outpatient care, non-cancer related	7,113	1,756	1,477	1,542	1,532
Chemotherapy, oral drugs	309	343	637	1,221	1,176
Non-cancer-related drugs	3,457	1,332	1,166	996	1,416
Total	81,565	60,492	59,308	52,774	53,203
Immuno-oncology drugs					
Number of patients at risk	1,436	871	532	351	193
Cost by healthcare resource type (€)					
ALK-targeted drugs	429	502	492	418	440
EGFR-targeted drugs	513	611	746	1,153	1,349
Immuno-oncology drugs	58,039	3,845	2,755	3,983	1,058
Immunosuppressants	495	62	40	45	36
Inpatient care, cancer related	9,439	4,050	3,006	1,785	1,584
Inpatient care, non-cancer related	16,103	4,969	4,551	3,649	4,083
Outpatient care, cancer related	7,160	4,243	3,045	1,636	911
Outpatient care, non-cancer related	8,864	1,894	1,782	1,610	1,683
Chemotherapy, oral drugs	604	686	1,147	1,138	1,170
Non-cancer-related drugs	5,061	1,423	1,367	1,101	1,028
Total	106,707	22,285	18,931	16,518	13,342
